# The Impact of Instant Reimbursement of Cross-Regional Medical Services on Hospitalization Costs Incurred by the Floating Population—Evidence from China

**DOI:** 10.3390/healthcare10061099

**Published:** 2022-06-13

**Authors:** Xiaodong Zhang, Lei Zhang

**Affiliations:** Institute of Population Research, Peking University, Beijing 100781, China; xiaodongzhang@stu.pku.edu.cn

**Keywords:** medical insurance, instant reimbursement, manual reimbursement, hospitalization costs, floating population

## Abstract

Background: The medical cost reimbursement function of medical insurance can reduce the medical burden of individuals and thus improve their medical service utilization level. This study aimed to explore the impact of different cross-regional reimbursement types of medical insurance (instant reimbursement and manual reimbursement) on the hospitalization costs incurred by the floating population. Methods: The data used in this study was from the 2018 China Migrants Dynamic Survey (CMDS) conducted by the National Health Commission of China. The multiple linear regression model and Propensity Score Matching method (PSM) were used to analyze the impact of instant and manual reimbursement on hospitalization costs. Results: (1) Instant reimbursement and manual reimbursement could significantly reduce the floating population’s out-of-pocket proportion of hospitalization costs by 33.2% and 26.9%, respectively; (2) the average proportion and amounts of out-of-pocket hospital costs of instant reimbursement for the floating population were lower than those of manual reimbursement by 6.35% and 19.6%, respectively, and this impact would gradually increase as the flow distance expanded; (3) there was no significant impact of instant reimbursement on the total hospitalization costs relative to manual reimbursement. Conclusions: Our study suggests that instant reimbursement can effectively reduce the out-of-pocket medical burden of the floating population at the individual level, but it will not have an obvious impact on the total hospitalization costs at the social level.

## 1. Introduction

**Figure 1:**
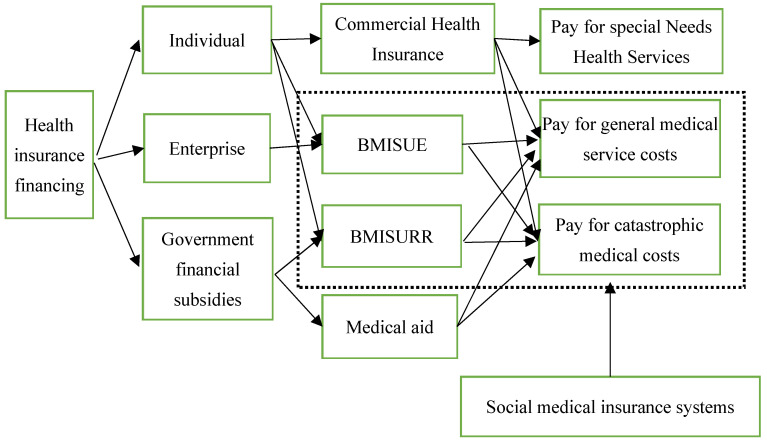
Framework of China’s medical insurance system.

Health is the most important component with regard to human capabilities and health equity has a special significance compared with other social equities [1]. In order to reduce the burden of medical care, improve the overall health status of the population, and promote health equity, the Chinese government launched a new round of healthcare reform in 2009. The primary goals of the reform are to expand the coverage of basic medical insurance and to improve the benefits of medical insurance and the management level of health services. The Chinese government established the Basic Medical Insurance System for Urban Employees (BMISUE) and the Basic Medical Insurance System for Urban and Rural Residents (BMISURR), which cover all residents (see Figure 1). The coverage and benefits level of medical insurance have been greatly improved. According to the data released by the National Health Insurance Administration of China in 2021, the Chinese government has almost achieved universal medical insurance coverage, with more than 1.36 billion people covered by basic medical insurance and a stable participation rate of over 95%. At the same time, the benefits of medical insurance have also improved significantly. The average reimbursement proportion of hospitalization costs for BMISUE and BMISURR reached 85.2% and 70.0%, respectively [2]. Previous studies found that the realization of universal medical insurance could significantly re- duce residents’ medical burden when seeking medical care and improve the utilization level of health services [3,4].

However, although the Chinese government has rapidly realized universal healthcare, the unique “localized management” principle of China’s medical insurance systems means that not every resident has the same access to convenient and affordable health services. The current medical insurance systems are mainly coordinated at the county or city level in China. The health insurance funds are raised, paid, and managed uniformly within the coordinated areas. In other words, China’s social medical insurance systems have strong regionality and non-portability. The reimbursement of medical insurance will be inconvenient for individuals when they seek medical treatment beyond the coordinated area. Under the framework of the “localized management” policy of medical insurance, the insured who seek cross-regional medical treatment must first obtain administrative approval from the healthcare security administration of the insured area, then visit the designated hospitals in their current residence and pay all the medical expenditures in advance, and finally return to the healthcare security administration of the insured area to handle a series of cumbersome reimbursement procedures. The duration of reimbursement is relatively long [5], which will increase the additional costs of patients (i.e., time costs and transportation costs). Some studies suggested that the “inconvenience” of cross-regional medical insurance reimbursement made it difficult for insured residents to enjoy timely, convenient, and affordable medical services when they work beyond the coordinated area of medical insurance, which may increase the medical burden of residents who seek cross-regional medical treatment, and was not conducive to the realization of health equity [6,7,8]. For example, Ren Y (2016) found that when cancer patients seek care beyond coordinated area of medical insurance, the reimbursement rates and coverage were relatively low, which could significantly increase their financial burden [9].

Most importantly, there is another reality that should not be overlooked. Large-scale and regular population mobility has become the “new normal” since the Reform and Opening-up. The increasing floating population will inevitably increase the demand for cross-regional medical services. According to the data from the National Bureau of Statistics, the floating population in China increased from 102.29 million in 2000 to 375.82 million in 2020, and the cross-provincial floating population increased from 37.23 million to 124.84 million, with an average annual increase of 13.25% and 11.75%, respectively [10]. In the context of large-scale population mobility, the inconvenience of cross-regional medical services reimbursement has further aggravated the inequity in health service utilization between the insured floating population and the local residents, resulting in a situation of “difficulty-and-high costs of getting medical services” for the floating population. Since the insured place of the floating population is inconsistent with their residence, the inconvenience of the cross-regional reimbursement will limit their choices of medical treatment, reduce their utilization of health services, and thus affect their health status. Numerous studies have found that the level of medical service utilization and the proportion of health insurance reimbursement of the floating population (especially for the cross-provincial floating population) was much lower than those of local residents [11,12,13,14,15,16].

In order to deal with the conflict between the restriction of the “localized management” of medical insurance and the accelerated population mobility, and thus to meet the increasing demand of cross-regional medical treatment for the floating population, the Chinese government carried out a drastic reform of the medical insurance system, and one of the most important measures was to conduct an instant reimbursement reform of cross-regional medical services. From 2009 to 2014, the instant reimbursement mechanism of cross-regional medical services was gradually established. It began with instant settlement of intra-provincial hospitalization costs, and then gradually shifted to cross-provincial. By the end of 2016, a national networking of social medical insurance and a national cross-regional instant settlement platform of medical expenditures had been established. Then, the cross-provincial instant settlement of outpatient costs became the core of the reform from 2017. Data from the National Health Insurance Administration showed that the level of residents’ health service utilization increased significantly. China’s annual cross-provincial instant settlement reached 4.459 million times in 2021. Total medical costs reached 1070.20 billion yuan, and nearly 60% was paid by the medical insurance fund [2].

The main aims of the cross-regional instant settlement of medical insurance are to reduce financial inaccessibility, improve the utilization level of health services of residents who seek cross-regional medical treatment, and thus promote health equity. However, does the reform of cross-regional instant settlement of medical insurance achieve the government’s expectations? Some scholars have explored the impact of the instant settlement of medical insurance on residents’ health service utilization levels and have concluded that cross-regional instant settlement could reduce medical costs and increase the medical service utilization of residents who seek cross-regional medical treatment [17,18]. For instance, He and Hou (2016) found that medical insurance participating in the place of household registrations suppressed residents’ health service utilization levels before the instant settlement policy was implemented, but this “suppression effect” quickly disappeared, and the residents’ health service utilization levels significantly improved when the government realized the instant reimbursement of cross-regional medical services [19]. Jin Y (2018) found that the implementation of the instant reimbursement policy of cross-regional medical services can promote social equity and further improve the medical services utilization level of participants [20]. Huang (2017) also discovered that the instant settlement of cross-provincial medical treatment promoted social equity and greatly reduced the financial burden of insured residents, especially for patients with relatively poor financial conditions [21].

However, some scholars have drawn opposite conclusion. They argued that instant settlement would change participants’ behavior and direction of medical treatment, which may cause a serious “siphon effect” in general hospitals and increase medical costs of cross-regional medical treatment [22]. Based on data retrieved from the instant settlement platform, some scholars compared the changes in medical costs after the implementation of instant settlement, and found that medical costs increased significantly [23,24]. Zhan et al. (2022) also found that choosing instant reimbursement significantly improved the total medical cost for children with leukemia [25]. Moreover, some scholars also compared the utilization difference of health services between local medical treatment and cross-regional medical treatment, and found that the reimbursement rate of health insurance for cross-provincial medical services was significantly lower than that of local residents, which increased the financial burden of cross-regional patients [26]. Zhou and Liu (2016) used a generalized linear model (GLM) to explore the impact of the Basic Medical Insurance System for Urban Residents (BMISUR) on healthcare services for the floating population and found that the medical insurance reimbursement level of the floating population was lower than that of local residents, which will suppress the floating population’s demand for formal health care services [13].

On the whole, the existing literature has either analyzed the impact of the instant settlement of medical insurance on residents’ health care costs and health service utilization, or compared the utilization differences of health services between the cross-regional instant settlement and local medical care. Few studies have explored the impact of different cross-regional medical insurance reimbursement types on residents’ healthcare costs in the process of utilizing cross-regional medical services. Currently, there are mainly two types of medical insurance reimbursement patterns for residents when seeking cross-regional medical treatment: instant reimbursement in their current residence and manual reimbursement in the insured place of household registration. This study aimed to examine the impact of different cross-regional medical insurance reimbursement patterns on the hospitalization costs incurred by the floating population, and further explored the potential impact mechanism.

This study aimed to compare the average out-of-pocket hospitalization cost differences between instant and manual imbursement and answer the question of whether the instant reimbursement of cross-regional medical treatment can reduce the average out-of-pocket hospitalization expenditure level of the floating population. Moreover, we compared the differences in the impacts of instant and manual reimbursement on the total hospitalization costs incurred by the floating population. Finally, considering that China’s current medical insurance systems are mainly coordinated at the county or city level, the social medical insurance systems have strong regionality and non-portability characteristics. The farther the flow distance, the stronger the regionality and non-portability of medical insurance, which will affect the floating population’s cross-regional medical services. We further explored whether flow distance impacts on the difference in hospitalization costs between instant and manual reimbursement.

## 2. Materials and Methods

### 2.1. Data

The data used in this study were retrieved from the latest China Migrants Dynamic Survey (CMDS), which was conducted by the National Health Commission of China in May 2018. The CMDS adopted the PPS method of stratified, multi-stage, and proportional to the population size for sampling, and the respondents consisted of those who were aged over 15, had lived in the destination for more than one month, and had a household registration beyond the local county or city. The survey covered basic household demographic characteristics, employment and consumption, medical security, basic public health services, and other modules. It is a nationally representative large-scale microscopic social survey database of the floating population in mainland China. A total of 152,000 floating population samples were collected in the 2018 CMDS data. 

**Figure 2:**
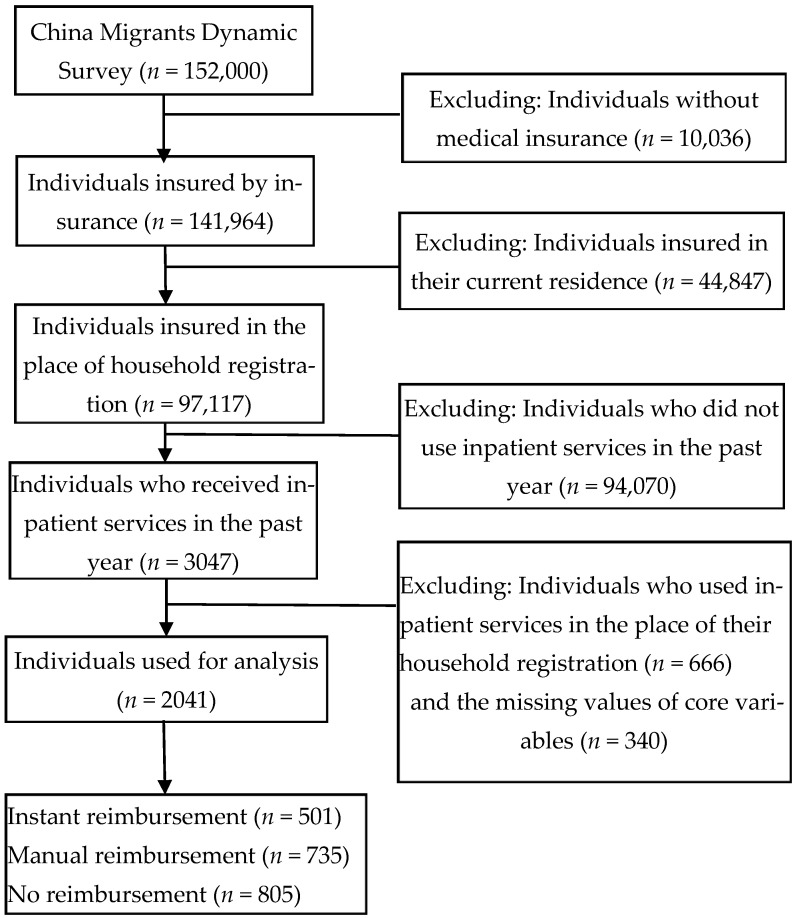
Flowchart of samples screening.

We cleaned the data in detail according to the research demands (see Figure 2). First, since the floating population who are insured in their current residence can enjoy convenient medical services and reimbursement in their current residence and there is no need for cross-regional reimbursement, we deleted observations who were insured in their current residence. Second, when a member of the floating population needs inpatient services, they can choose to use hospitalization services in their current residence or return to the insured place of household registration. For those who return to their insured place of household registration for hospitalization, they can enjoy medical insurance reimbursement directly in the insured place of household registration, and there is no cross-regional reimbursement demand. Therefore, we excluded the observations of the floating population who returned to their insured place of household registration for hospitalization services and only retained the observations of those who received inpatient services in their current residence in the past year. Finally, after removing the observations without any medical insurance and with missing values of core variables and extreme values of income, the final observations used for our analysis were 2041. We used these observations to analyze the impact of different cross-regional reimbursement types on the hospitalization costs incurred by the floating population.

### 2.2. Variable Measurement

#### 2.2.1. Dependent Variables: Medical Costs Status of the Floating Population

Previous studies measured residents’ medical expenditures mainly through out-of-pocket medical expenditures, out-of-pocket proportion of medical expenditures, per capita medical costs, and inpatient medical expenditures [3,27,28]. According to previous studies and the questionnaire design of the 2018 CMDS, this paper mainly used the average proportion and amounts of out-of-pocket hospitalization costs and the total hospitalization costs to measure the medical expenditure level of cross-regional medical treatment. Specifically, we used the survey question “What was the total cost of your last hospitalization?” and “How much did you/your family eventually pay out-of-pocket for the total costs of the last hospitalization (excluding reimbursement and personal medical account expenses)?” to measure the total costs and out-of-pocket costs incurred by the floating population, respectively. The out-of-pocket proportion of hospitalization indicator was obtained by dividing out-of-pocket costs by total hospitalization costs. 

#### 2.2.2. Independent Variables: The Reimbursement Types of Cross-Regional Medical Services for the Floating Population

The core independent variable of this study was the types of medical insurance reimbursement. Currently, there are mainly two reimbursement choices of medical insurance when the floating population utilizes cross-regional inpatient services: instant reimbursement in the destination (instant reimbursement) or manual reimbursement in their insured place (manual reimbursement). To compare the impact of different reimbursement choices on the hospitalization costs, we constructed a dummy variable of “Reimbursement”. 

#### 2.2.3. Controlled Variables

In order to reduce the possible estimation deviation in the statistical model due to missing variables, three types of variables were controlled in our analysis, which were demographic indicators (including age, gender, household registration, ethnic status, family size, and flow distance), the human capital status (including education level and self-rated health status), and socioeconomic status indicators (including the family monthly income (logarithm) and the types of work). Considering that the regional differences in the economic development in current China, which may have an impact on the floating population’s hospitalization costs, we controlled for the regional differences as well. The definitions and descriptive results of above variables are shown in Table 1.

### 2.3. Model

#### 2.3.1. Multiple Regression Model

Among the indicators measuring the utilization of hospitalization service, the average out-of-pocket proportion and amounts of hospitalization costs, and the total hospitalization costs are continuous variables. Therefore, we adopted a multiple linear regression model to estimate the impact of instant reimbursement of cross-regional medical services on the hospitalization costs incurred by the floating population. The model is

(1) $$Hospitalization\;costs_{i}=\beta_{0}+\beta_{1}Reimbursements_{i}+\beta_{2}X_{i2}\ldots \beta_{k}X_{ik}+\varepsilon_{i}$$

where $Hospitalization\;costs_{i}$ represents the level of hospitalization costs incurred by the ith member of the floating population, $Reimbursement_{i}$ represents the types of cross-regional reimbursement chosen by the ith respondent, and $X_{2}\ldots X_{k}$ represent the other controlled variables, including the demographic variables, human capital status variables, and the socioeconomic status indicators that may affect the level of hospitalization costs. $\beta_{0}$ is the intercept term, $\beta_{1}$ is the correlation coefficient matrix of the impact of reimbursement patterns on the hospitalization costs, $\beta_{2}\ldots \beta_{k}$ are the correlation coefficients of other control variables on the medical costs incurred by the floating population, and $\varepsilon_{i}$ is a random error term.

#### 2.3.2. Propensity Score Matching Model (PSM)

Since the hospitalization behavior of the floating population is highly self-selective, we were unable to identify the sample who should have received hospitalization but did not actually use hospitalization services. In this case, the estimation of the regression results is biased due to sample selection deviation. To reduce the possible selective deviation in the sample, we used the Propensity Score Matching method (PSM) to analyze the effect of different reimbursement types on hospitalization costs. Theoretically, the PSM model can reduce the selective deviation caused by confounding factors and overcome the potential endogenous problem in the model. The core idea of matching estimation is to find the samples within the control group similar to the samples in the treated groups, so as to identify the counterfactual individuals of the treatment group. In this case, we can achieve unbiased and consistent estimation [29,30]. There are mainly four steps in PSM analysis: calculating the propensity values using logit models, conducting score matching, evaluating the balance after matching, and calculating the average treatment effect (ATT). In this study, we adopted K-nearest neighbor matching (one-to-four), caliper matching, and kernel matching. Specifically, after matching based on propensity scores, the average treatment effect (ATT) for floating population can be expressed as

$$\mathrm{ATT}=E\left(Y_{1}|p=1\right)-E(Y_{0}|p=1)$$

where $Y_{1}$ denotes the average value of the explanatory variable when the treated group sample receives the intervention, and $Y_{0}$ denotes the average value of the explanatory variable when the treated group sample is assumed not to receive the intervention. The former represents the average hospitalization costs of floating population who chose instant reimbursement, and the latter represents the average hospitalization costs if those floating population had opted for manual reimbursement. Since the latter cannot be directly observed, the counter-factual estimation is required, and $E(Y_{0}|p=1$) is the counter-factual effect.

## 3. Results

### 3.1. Descriptive Analysis

We first report the mean differences of hospitalization costs between instant and manual reimbursement. Table 2 shows the results of the *t*-test. The results indicated that there were significant differences in the hospitalization costs between instant and manual reimbursement (*p* < 0.001).

### 3.2. The Impact of Different Cross-Regional Reimbursement Types on the Hospitalization Costs Incurred by the Floating Population

We further used the least squares estimation to examine the impact of different cross-regional reimbursement types on the hospitalization costs of the floating population. Column (1) of Table 3 categorized the reimbursement status of hospitalization costs into “reimbursed by medical insurance” and “having no reimbursement”, and took “having no reimbursement” as the benchmark variable. The regression results suggested that medical insurance reimbursement could significantly reduce the floating population’s out-of-pocket proportion of hospitalization costs by 30.1% (*p* < 0.01). Column (2) categorized the floating population’s medical insurance reimbursement into “instant reimbursement”, “manual reimbursement”, and “having no reimbursement” to identify the impact of medical insurance reimbursement types on the hospitalization costs. It suggested that the out-of-pocket proportion of instant reimbursement and manual reimbursement were lower than those without any reimbursement by 33.2% (*p* < 0.001) and 26.9% (*p* < 0.001), respectively.

Column (3) excluded the samples without reimbursement and reported the potential difference between instant and manual reimbursement. The regression results suggested that instant reimbursement could significantly reduce the out-of-pocket proportion of hospitalization costs by 6.35% relative to manual reimbursement (*p* < 0.001). Column (4) further compared the impact of instant and manual reimbursement on the floating population’s out-of-pocket hospitalization costs. The results suggested that the out-of-pocket hospitalization costs of instant reimbursement were lower than of manual reimbursement by 19.6% (*p* = 0.016). Column (5) reported the impact of instant reimbursement on the total hospitalization costs. The results showed that the instant reimbursement had a negative impact on the total hospitalization costs incurred by the floating population relative to manual reimbursement, but it was not statistically significant. 

### 3.3. Selection Bias: A Further Analysis Based on the PSM Method

An analysis of Table 3 revealed that both instant and manual reimbursement could significantly reduce the level of out-of-pocket hospitalization costs when receiving cross-regional medical services. However, there were obvious differences between instant and manual reimbursement with respect to the hospitalization costs. Table 4 further reported the PSM results for the impact of instant and manual reimbursement on the hospitalization costs. Column (1) of Table 4 reported the impact of instant and manual reimbursement on the out-of-pocket proportion of hospitalization costs. The results showed that the ATT coefficients for K-nearest neighbor matching, caliper matching, and kernel matching were −0.0636, −0.0611, and −0.0665, respectively. The ATT coefficients were consistent with the regression coefficient of Column (3) in Table 3, which indicated that the instant reimbursement could significantly reduce the out-of-pocket proportion of costs relative to those who chose manual reimbursement. Column (2) reported the impact of instant and manual reimbursement on the amounts of out-of-pocket costs, and the results indicated that the instant reimbursement can significantly reduce the medical burden of the floating population by reducing their out-of-pocket costs. Column (3) reported the impact of instant reimbursement of cross-regional medical services on total hospitalization costs, and the results suggested the instant reimbursement had no significant impact on total hospitalization costs. There was no significant difference in total hospital costs between instant and manual reimbursement.

### 3.4. The Impact of Cross-Regional Medical Services Reimbursement on the Hospitalization Costs under Different Flow Distances

Table 5 divided the flow distance of the floating population into three types: “cross-provincial flow”, “cross-city within the province”, and “cross-county within the city”. And reported the impact of the instant reimbursement on the hospitalization costs under different flow distances. The results suggested that, relative to manual reimbursement, the instant reimbursement could reduce the proportion and amounts of out-of-pocket hospitalization costs incurred by the cross-provincial floating population by 11.1% and 35.9%, respectively. However, in terms of total hospitalization costs, there was no significant effect between these two reimbursement types under different flow distances.

## 4. Discussion

Previous studies mainly analyzed the differences in health care utilization levels between patients who received cross-regional medical services and local residents, and few of them examined the impact of different reimbursement types of cross-regional medical services on the medical costs of floating populations. Based on the 2018 CMDS data, this study empirically analyzed the impact of the instant reimbursement of cross-regional medical treatment on hospitalization costs incurred by the floating population in the context of normalized population mobility in China. Our study found the following: (1) Instant and manual reimbursement can significantly reduce the floating population’s out-of-pocket proportion of hospitalization costs by 33.2% and 26.9%, respectively. (2) There were obvious hospitalization utilization differences between instant and manual reimbursement. The average proportion and amounts of out-of-pocket costs of instant reimbursement were lower than those of manual reimbursement by 6.35% and 19.6%, respectively. (3) Instant reimbursement had no impact on the total hospitalization costs relative to manual reimbursement. (4) The impact of instant reimbursement on the average proportion and amounts of out-of-pocket hospitalization costs would gradually increase as the flow distance expanded, with the greatest impact on the cross-provincial floating population.

Social medical insurance, as an important disease-risk-sharing mechanism, can improve patients’ health care utilization by increasing their financial accessibility when the occurrence of disease risks [27,31]. Our study found that this disease-risk-sharing function of health insurance still existed when the floating population sought cross-regional medical treatment. Both instant and manual reimbursement could greatly reduce the average out-of-pocket proportion of hospitalization costs, which was consistent with the findings of existing studies showing that medical insurance can reduce the level of out-of-pocket medical costs and increase the level of preventive care and medical service utilization for individuals [4,12,32,33]. Our results shown that the floating population could obtain a certain proportion of medical cost reimbursement regardless of which medical insurance reimbursement types they chose, so the medical burden of the floating population could be reduced in the process of cross-regional medical treatment.

Moreover, we found that instant and manual reimbursement had different impacts on the hospitalization costs. The average proportion and amounts of out-of-pocket hospitalization costs of instant reimbursement were lower than those of manual reimbursement, and this effect was greater for the cross-provincial floating population. Before the implementation of instant reimbursement reform, the floating population could only choose to return to the insured place for manual reimbursement when they receive medical treatment at the designated medical institutions. The reimbursement ratio was relatively low, and the reimbursement procedures were cumbersome, which increased their out-of-pocket medical costs and thus reduced the level of their medical service utilization [34]. Since the instant reimbursement reform, the floating population now only needs to register at the Healthcare Security Administration of the insured place through the Internet or by telephone, and subsequently bring their social security card to the designated hospital to conduct instant reimbursement in the local medical institution. The level of benefits is consistent with the insured place, which, by increasing the convenience of cross-regional medical insurance reimbursement directly, breaks the localized management restriction of medical insurance and greatly improves the utilization level of medical services [35,36]. More importantly, the instant reimbursement can greatly save time, transportation costs, and debit costs of the floating population, contributing to more convenient access to medical service resources for those who seek cross-regional medical treatment [17].

Finally, we found that, although instant reimbursement compared with manual reimbursement could significantly reduce the floating population’s average proportion and amounts of out-of-pocket costs, there were no obvious differences in total hospitalization costs, which was inconsistent with the findings of existing studies. With the implementation of the instant reimbursement reform, the arguments about the rapid rise of total medical costs and the sustainability of medical insurance funds have been the focus of scholars’ attention. Previous studies have drawn two different conclusions regarding the impact of the instant reimbursement reform on the total medical costs. Some scholars believed that, since the Healthcare Security Administration of the insured place failed to effectively monitor the behavior of cross-regional medical institutions, patients of insured places were more likely to suffer from the risk of excessive medical treatment under the condition of information asymmetry between doctors and patients, which might cause an unreasonable increase in cross-regional medical costs [37,38,39,40]. Furthermore, some scholars believed that the instant settlement policy might change the medical treatment behavior and direction of participants, and aggravate the “siphoning effect” of general hospitals. As a consequence, the medical costs of cross-regional medical treatment increase rapidly [22]. Cui (2021) studied the differences in medical costs after the implementation of the instant settlement and found total medical costs increased significantly [24].

Another study suggested that instant reimbursement could generate a significant “cost-control effect”. The hospitalization costs of cross-regional instant reimbursement are lower than those of manual reimbursement, since the Healthcare Security Administration of the insured place can regulate the behavior of cross-regional medical treatment through real-time monitoring, so any unnecessary waste of medical resources can be avoided [41]. In contrast, our study found that there were no significant difference between instant and manual reimbursement in terms of total hospital costs, which means that the instant reimbursement neither has a “cost-control effect” nor leads to a further increase in total medical costs. We argue that the core reason for this is that the level of coordination of the medical insurance is too low. Currently, China’s medical insurance systems are mainly coordinated at the county or city level, and the medical insurance policy is relatively independent within the coordinated area. As a result, the level of medical insurance funding, the proportion of reimbursement, the deductible line, and the healthcare directory of medical insurance vary among different coordinated areas. In the context of cross-regional instant reimbursement, the government requires the patients use medical treatment according to the healthcare directory of their local health department and enjoy the reimbursement benefits according to the reimbursement policy of the insured place. In this case, even though the Chinese government has achieved instant reimbursement reform, the medical insurance agencies of insured places are still unable to effectively supervise the cross-regional medical institutions. A direct consequence of this is that the cost-control effect of instant settlement is not obvious. In fact, even if there is no cross-regional medical practice, the medical costs will inevitably rise because of the universal “information asymmetry” problem and “siphoning effect” of general hospitals in the medical service market. Therefore, raising the coordination level of basic medical insurance, carrying out comprehensive hierarchical diagnosis and treatment, and exploring various medical insurance payment methods are needed to control the rapid rise of medical costs in the future.

## 5. Conclusions and Limitations

This study empirically analyzed the impact of the different cross-regional reimbursement types of medical insurance on hospitalization costs of the floating population. Our study found that both instant and manual reimbursement can significantly reduce the floating population’s out-of-pocket proportion of hospitalization costs, but there were obvious hospitalization utilization differences between these two types. The average proportion and amounts of out-of-pocket hospital costs of instant reimbursement were lower than those of manual reimbursement, and this impact would gradually increase as the flow distance expanded. Finally, instant reimbursement had no impact on the total hospitalization costs relative to manual reimbursement. Our study suggested that instant reimbursement could effectively reduce the out-of-pocket medical burden of the floating population at the individual level, but it might not have an impact on the total hospitalization costs.

There are some limitations in this study. Firstly, the Chinese government has fully realized the cross-provincial instant reimbursement of hospitalization costs, and the cross-provincial instant reimbursement of outpatient costs also remains in the pilot stage. Unluckily, the latest CMDS data only provides the inpatient costs incurred by the floating population. Therefore, this study fails to explore the potential impact of instant reimbursement on outpatient costs due to data limitations. Furthermore, the varieties of disease and durations of hospitalization may have vital impacts on hospitalization costs for floating populations. In order to minimize the estimation bias, we controlled the health status of floating population and used the PSM method to overcome the potential missing variables. However, we still cannot obtain the net effect of the variety of diseases and hospitalization durations on the hospitalization costs between instant and manual reimbursement due to data limitations. Finally, compared to the original data obtained directly from hospitals, there are some errors in using social survey data in this study. We will conduct relevant research once the data become available.

## Figures and Tables

**Table 1:** Variable definition and descriptive results.

**Variables**	**Mean**	**SE**
The average out-of-pocket proportion of hospitalization costs	0.707	0.289
The average amounts of out-of-pocket hospitalization costs	8.479	1.048
Total hospitalization costs	8.923	0.969
Age	40.29	15.169
Family size	3.45	1.164
Monthly household income	8.628	0.923
**Variables**	**Frequency**	**Percent (%)**
The type of reimbursement		
Having no reimbursement	805	39.44
Instant reimbursement	501	24.55
Manual reimbursement	735	36.01
Gender		
Male	688	33.71
Female	1353	66.29
Ethnic status		
Han	1804	88.39
Minority	237	11.61
Household registration		
Rural	1371	67.17
City/Town	670	32.83
The flow distance		
Cross-provincial mobility	732	35.86
Intra-provincial mobility	1309	64.14
Education level		
Illiteracy/Elementary school	481	23.57
Middle school	836	40.96
High school/Vocational school	438	21.46
Three-year college and above	286	14.01
Self-rated health		
Very good	1158	56.73
Good	517	25.33
Fair	343	18.81
Poor	23	1.13
Health records		
Yes	570	27.93
No	1471	72.07
Types of work		
Having no work	1089	53.35
Government institution/State-owned enterprises/Collective enterprise/Private enterprise/The joint venture enterprise	292	14.31
Individual Businesses	492	24.11
Others	168	8.23
Regional differences		
Eastern provinces	672	32.92
Central provinces	469	22.99
Western provinces	752	36.84
Northeastern province	148	7.25

**Table 2:** The mean differences of hospitalization costs incurred by the floating population between instant and manual reimbursement.

Variables	Instant Reimbursement (*n* = 501)	Manual Reimbursement (*n* = 735)	*p*
The average out-of-pocket proportion of hospitalization costs	0.536 (0.223)	0.618 (0.232)	<0.001
The average out-of-pocket hospitalization costs	8.268 (1.036)	8.559 (1.051)	<0.001
Total hospitalization costs	8.995 (0.874)	9.131 (0.889)	<0.001

**Table 3:** The regression results for the impact of instant reimbursement on the hospitalization costs of the floating population.

	(1)		(2)		(3)		(4)		(5)	
Variables	The Average Proportion of Out-of-Pocket Hospitalization Costs		The Average Proportion of Out-of-Pocket Hospitalization Costs		The Average Proportion of Out-of-Pocket Hospitalization Costs		The Average Amounts of Out-of-Pocket Hospitalization Costs		Total Hospitalization Costs	
	Coefficient	*p*	Coefficient	*p*	Coefficient	*p*	Coefficient	*p*	Coefficient	*p*
Reimbursement	−0.301	<0.001								
Instant reimbursement			−0.332	<0.001	−0.0635	<0.001	−0.196	0.016	−0.072	0.743
Manual reimbursement			−0.269	<0.001						
Other variables	Controlled		Controlled		Controlled		Controlled		Controlled	
Regional differences	Controlled		Controlled		Controlled		Controlled		Controlled	
Observations	2041		2041		1236		1236		1236	
R-squared	0.338		0.332		0.111		0.044		0.104	

**Table 4:** PSM results for the impact of instant and manual reimbursement on the hospitalization costs.

	Hospitalization Costs	Matching Types	Treated	Controlled	Difference	S.E	*p*
(1)	Out-of-pocket ratio of hospitalization costs	K-nearest neighbor matching	0.5359	0.5995	−0.0636	0.0157	<0.001
		Caliper matching	0.5352	0.5963	−0.0611	0.0148	<0.001
		Kernel matching	0.5359	0.6024	−0.0665	0.0144	<0.001
(2)	Out-of-pocket hospitalization costs	K-nearest neighbor matching	8.2016	8.4161	−0.2145	0.0937	0.021
		Caliper matching	8.1972	8.4024	−0.2052	0.0869	0.019
		Kernel matching	8.2016	8.4265	−0.2249	0.0845	0.023
(3)	Total hospitalization costs	K-nearest neighbor matching	8.9952	9.0899	−0.0947	0.0670	0.168
		Caliper matching	8.9978	9.0831	−0.0853	0.0657	0.186
		Kernel matching	8.9952	9.0195	−0.0963	0.0611	0.165

Note: The samples that had no medical insurance reimbursement were excluded. The treatment group was “instant reimbursement” and the control group was “manual reimbursement”.

**Table 5:** The regression results for the impact of cross-regional reimbursement of medical services on the hospitalization costs under different flow distances.

	(1)		(2)		(3)		(4)		(5)		(6)		(7)		(8)		(9)	
	The Proportion of Out-of-Pocket Hospitalization Costs						The Out-of-Pocket Hospitalization Costs						Total Hospitalization Costs					
Variables	Cross-Provincial Flow		Cross-City within the Province		Cross-County within the City		Cross-Provincial Flow		Cross-City within the Province		Cross-County within the City		Cross-Provincial Flow		Cross-City within the Province		Cross-County within the City	
	Coefficient	*p*	Coefficient	*p*	Coefficient	*p*	Coefficient	*p*	Coefficient	*p*	Coefficient	*p*	Coefficient	*p*	Coefficient	*p*	Coefficient	*p*
Instant reimbursement	−0.111	0.003	−0.060	0.011	−0.027	0.205	−0.359	0.032	−0.147	0.163	−0.088	0.376	−0.127	0.375	−0.037	0.687	−0.030	0.732
Control variables	Controlled		Controlled		Controlled		Controlled		Controlled		Controlled		Controlled		Controlled		Controlled	
Observations	324		495		417		324		495		417		324		495		417	
R-squar	0.131		0.126		0.176		0.111		0.014		0.090		0.151		0.128		0.101	

## Data Availability

The datasets generated for this study are available on request to the corresponding author.
